# Antibacterial Activity of Partially Oxidized Ag/Au Nanoparticles against the Oral Pathogen *Porphyromonas gingivalis* W83

**DOI:** 10.1155/2016/9605906

**Published:** 2016

**Authors:** Megan S. Holden, Jason Black, Ainsely Lewis, Marie-Claire Boutrin, Elvin Walemba, Theodore S. Sabir, Danilo S. Boskovic, Aruni Wilson, Hansel M. Fletcher, Christopher C. Perry

**Affiliations:** 1 Division of Biochemistry, Loma Linda University School of Medicine, Loma Linda, CA 92350, USA; 2 Northern Caribbean University, Manchester, Jamaica; 3 Division of Microbiology and Molecular Genetics, Loma Linda University School of Medicine, Loma Linda, CA 92350, USA; 4 Department of Earth and Biological Sciences, Loma Linda University School of Medicine, Loma Linda, CA 92350, USA; 5 College of Arts and Sciences, Faulkner University, Montgomery, AL 36109, USA

## Abstract

Advances in nanotechnology provide opportunities for the prevention and treatment of periodontal disease. While physicochemical properties of Ag containing nanoparticles (NPs) are known to influence the magnitude of their toxicity, it is thought that nanosilver can be made less toxic to eukaryotes by passivation of the NPs with a benign metal. Moreover, the addition of other noble metals to silver nanoparticles, in the alloy formulation, is known to alter the silver dissolution behavior. Thus, we synthesized glutathione capped Ag/Au alloy bimetallic nanoparticles (NPs) *via* the galvanic replacement reaction between maltose coated Ag NPs and chloroauric acid (HAuCl_4_) in 5% aqueous triblock F127 copolymer solution. We then compared the antibacterial activity of the Ag/Au NPs to pure Ag NPs on *Porphyromonas gingivalis* W83, a key pathogen in the development of periodontal disease. Only partially oxidized glutathione capped Ag and Ag/Au (Au:Ag≈0.2) NPs inhibited the planktonic growth of *P. gingivalis* W83. This effect was enhanced in the presence of hydrogen peroxide, which simulates the oxidative stress environment in the periodontal pocket during chronic inflammation.

## 1. Introduction

Advances in nanotechnology provide opportunities for the fabrication of silver containing nanoparticles (NPs) that can act as antimicrobial agents [1]. These NPs offer advantages over traditional therapies including reduced toxicity, lower cost, weak ability of bacteria to develop resistance [2, 3], and the ability to inhibit biofilms [4–6]. Additionally, silver (Ag) NPs [7] were shown to be less toxic to humans than traditional antibacterial Ag compounds such as silver nitrate [8] and silver sulfadiazine [9]. The physicochemical properties (size, shape, composition, and surface chemistry) of Ag NPs are known to influence the magnitude of their toxicity by affecting the degree of dissolution and delivery of silver ions [10–12]. Thus, the manipulation of these properties presents potential avenues for useful antibacterial preparations.

A potential application of Ag nanomaterials is to supplement or replace conventional antibiotic treatments for periodontal disease. Periodontal disease is characterized by tissue damage and subsequent tooth loss. Moreover, the most common form of periodontal disease, chronic periodontitis, occurs in nearly half of the Americans over the age of 30 [13,14]. Infection-induced periodontal disease is acknowledged to be polymicrobial in nature [15, 16] with the key pathogens, “the red complex,” being anaerobes [17, 18]. One of these “key pathogens” is *Porphyromonas gingivalis*, a black-pigmented gram-negative anaerobe. This anaerobe resides within the low oxygen environment of the periodontal pocket [19] and is implicated in manipulating the host immune system and bringing about changes in the oral microbial community that contribute to chronic inflammation and tissue damage [20]. Chronic inflammation results in an excess of oxidative species such as hydrogen peroxide (H_2_O_2_), superoxide radicals (^∙^O_2_^–^)and hydroxyl radicals (^∙^OH). Continuously elevated levels of oxidative species are known to cause oxidative damage to tissues and is termed “oxidative stress” [21, 22]. Moreover, the chronic inflammation associated with periodontal diseases is also a risk factor for cardiovascular diseases [23], diabetes [24], and rheumatoid arthritis [25, 26]. Thus, targeting pathogens in “the red complex” could be beneficial for the treatment of periodontal disease and for reducing the risk of occurrence of secondary diseases that are associated with it.

Conventional antibiotic therapeutic strategies against periodontal pathogens suffer from microbial resistance [27–29] and problems maintaining a functional effective dose within the periodontal pocket [29, 30]. Therefore, there is a need for antimicrobials that are effective against anaerobes, can remove mature oral biofilms, carry low risk of resistance building in bacteria, and have minimal or no side effects. Several studies have investigated the antibacterial effects of Ag NPs. The proposed mechanisms of Ag NP-related toxicity include membrane damage, reactive oxygen species-based lipid and DNA damage, Ag^+^ ion-based DNA damage, and interaction of Ag ions with intracellular proteins [31–33]. Nonetheless, the antibacterial mode of action is dependent upon the aqueous solution environment [34, 35] and the composition of the nanomaterial [36]. While Ag NPs are strongly antibacterial towards aerobes, such effects are greatly reduced or lost in anaerobic environments. Lok and coworkers showed that partially oxidized silver nanoparticles had antibacterial activity but zero-valent nanoparticles did not [37]. Alvarez and coworkers found that exposing Ag NPs to oxygen significantly enhanced NP toxicity towards *E. coli* under anaerobic conditions in a concentration dependent manner [10, 38]. Moreover, Ag NPs synthesized and tested under anaerobic conditions lacked antibacterial activity [10]. Lu and coworkers, conversely, reported that Ag NPs had some antibacterial activity against oral anaerobic bacteria [7]. However, they noted that the Ag NPs were more toxic to aerobic bacteria than to anaerobic bacteria and concluded that the enhanced toxicity under aerobic conditions was from oxygen-induced dissolution of silver ions (Ag^+^) from the Ag NPs [7]. In the case of eukaryotic cells, Prasad and coworkers found similar *in vitro* cellular stress responses after Ag NP and AgNO_3_ exposures and concluded that the oxidative stress and inflammation effects of Ag NPs are caused by Ag^+^ cytotoxicity [39].

It was further reported that the addition of other noble metals to silver nanoparticles, in the alloy formulation, is known to alter the silver dissolution behavior and reduce their overall toxicity towards eukaryotes [40]. Here we document a facile synthesis of Ag/Au biocompatible bimetallic NPs *via* the galvanic replacement reaction between maltose coated Ag NPs and chloroauric acid (HAuCl_4_) in 5% aqueous triblock F127 copolymer solution. This provides an alternative “green” approach to synthesize biocompatible Ag/Au NPs that are less cytotoxic toward eukaryotic cells than Ag NPs [41, 42]. The nanoparticles were capped with glutathione. *In vivo* studies have shown that glutathione capped gold nanoparticles do not produce morbidity and have increased biocompatibility, higher cellular uptake, and longer circulation times than polyethylene glycol (PEG) [43].

We present results showing the antibacterial effectiveness of silver and silver/gold bimetallic nanoparticles against the anaerobic oral pathogen *P. gingivalis* W83. Since oxidative stress is one of the main causes of the inflammatory environment found in the periodontal pocket, its physiological level has been simulated by the addition of hydrogen peroxide *in vitro* [21, 22, 44–48]. So in this study we also determined the effects of (0.1–0.25 mM) hydrogen peroxide induced stress on the antibacterial activity of Ag and Ag/Au nanoparticles against *P. gingivalis* W83.

## 2. Materials and Methods

### 2.1. Materials.

Ammonium hydroxide (28–30%), D-maltose (≥99%), NaOH (≥98%), silver nitrate (≥99%), gold(III) chlo-ride hydrate (HAuCl_4_·3H_2_O; 99.999% trace metals basis), Pluronic F127 (EO_100_PO_65_EO_100_, MW ≈ 12500; batch number 119K0073), and reduced glutathione were used as received (Sigma-Aldrich, Milwaukee, WI). Milli-Q water (Millipore) was used in all experiments.

### 2.2. Synthesis and Characterization of Nanoparticles.

Nano-particles were prepared in an anaerobic chamber (37°C) or aerobically (on the bench) at room temperature. Aerobically prepared NPs were stored under anaerobic conditions within 30 minutes of synthesis until use. Both Ag and Ag/Au NPs were characterized by atomic force (AFM) and electron microscopies as well as UV-vis, Fourier transform infrared spectroscopy (FTIR), and dynamic light scattering (DLS) (Supporting Information Section A in Supplementary Material available online at http://dx.doi.org/10.1155/2016/9605906). Maltose coated Ag NPs were prepared via the reduction of AgNO_3_ by maltose in an alkaline environment [49–51]. Bimetallic Ag/Au NPs were synthesized *via* the galvanic replacement reaction between maltose coated Ag NP seeds and HAuCl_4_ [51].

The concentrations of the Ag NPs were determined by interpolating the extinction coefficients. These functions are as follows: λ_max_ (*x*) = *a* + *b* · *x*^2^ (*a* = 397 nm; *b =* 9.58× 10^−3^ nm^−1^) The ε(λ_max_) in the size range 10–30 nm: *y*(*x*) = y_0_ + A · exp(*R* · *x*) (*y*_0_ = −1.43× 10^9^, *A =* 6.984× 10^8^, and *R* = 0.104), and between 30 and 100 nm: *y*(*x*) = *y*_0_ + *k* · *x* (*y*_0_= −4.709× 10^10^, *k* = 2.017 × 10^9^), where *x* is NP diameter[52]. The synthesized Ag NPs (≈3 nM,~10^12^ particles/mL), in 5% (w/v) F127, were diluted to an optical density of 12 at ≈400 nm by further addition of 5% Pluronic F127. Bimetallic NPs of different Ag: Au ratios were synthesized by adding HAuCl_4_ (0.2 M, 1 *μ*L or 0.1 M, 1–6 *μ*L) to 1 mL of Ag NP seed solution. The reaction was allowed to run for 30 minutes (the color change was instantaneous on thorough mixing) and the Au: Ag ratio was interpolated from the wavelength maxima and verified by electron-dispersive X-ray spectroscopy (EDS) (Figure S1). Material characterization of Ag/Au bimetallic NPs by transmission electron microscopy (TEM) is described elsewhere [51].

Silver and Ag/Au NPs were capped with glutathione by two methods. In method I, nanoparticles are capped with glutathione by adding 10 *μ*L of 10 mM glutathione (final concentration 0.1 mM) to 990 *μ*L of as-prepared Ag/Au nanoparticle solution. The reaction is allowed to run for 30 minutes and the resulting particles are diluted to OD_λmax_ = 1 in 5% Pluronic F127 and stored anaerobically. In method II, glutathione (0.1 mM, 10 *μ*L) was added to Ag and Ag/Au NPs in 1 mL 5% w/v F127 and incubated for ~12 hours prior to use. All NPs were stored under anaerobic conditions until use. To evaluate colloidal stability, 10 *μ*L of 10 mM glutathione (final concentration 0.1 mM) was added to 990 *μ*L of as-prepared nanoparticle solutions and the reaction was allowed to run for 30 minutes. The NPs were centrifuged and suspended in 5% aqueous F127 solution. These NPs were diluted 10-fold in 10 mM phosphate buffer saline solution (138 mM NaCl, 3 mM KCl). The extent of NP aggregation was assessed by DLS.

### 2.3. Bacterial Strain and Culture Conditions.

*P. gingivalis* W83 was grown in cysteine-free Brain Heart Infusion (BHI) br oth (Difco Laboratories, Detroit, MI) supplemented with yeast extract (5 mg/mL), hemin (5 *μ*g/mL), and vitamin K (0.5 *μ*g/mL) as per standard protocol for hydrogen peroxide stress experiments [44–48]. All cultures were incubated at 37°C unless otherwise stated. The *P*. *gingivalis* W83 strain was maintained in an anaerobic chamber (Coy Manufacturing, Ann Arbor, MI) in 10% H_2_, 10% CO_2_, and 80% N_2_. Growth rates for *P. gingivalis* W83 were determined spectrophotometrically (optical density at 600 nm) using a quartz cell with a 1 cm path length at 25°C.

### 2.4. NP Sensitivity and Survival Assays.

Overnight cultures of *P. gingivalis* W83 were used to inoculate prewarmed (37°C) BHI broth to early log phase (OD_600nm_ ≈ 0.1nm). These cultures were then split into equal aliquots (4.5–4.6 mL), incubated anaerobically at 37°C until OD_600nm_ ≈ 0.15 nm , and inoculated with either (a) 400 *μ*L sterile water, (b) 500 *μ*L stock NPs (0.03 nM final concentration), (c) 400 *μ*L stock NPs that were previously adjusted to OD = 1 or 4 (0.04–0.14 nM final concentration), or (d) 400 *μ*L 6.25 mM AgNO_3_ (0.5 mM final concentration), to give a 5 mL final volume. Inoculation of the controls with 400 *μ*L sterile water was done since the NP suspensions are aqueous. In separate experiments (data not shown), the growth of the bacteria in undiluted broth was comparable to inoculation with 400 *μ*L sterile water, inoculation with 400 *μ*L of 5% F127, and inoculation with 400 *μ*L glutathione (0.1 mM final concentration). The final dilution of the NPs resulted in an acceptable scattering error contribution (5–10%) for the UV-vis absorbance (OD_λmax_ < 0.1).

For H_2_O_2_ sensitivity and survival assays, overnight cultures were used to inoculate prewarmed (37°C) BHI broth to early log phase (OD_600nm_ ≈ 0.1nm ). These cultures were then split intoequal aliquots (4.5–4.6 mL), incubated anaerobically at 37°C until OD_600nm_ ≈ 0.20 nm , inoculated with anaerobically prepared NPs, and stressed with subinhibitory (0.1 mM) and inhibitory (0.25 mM) concentrations of H_2_O_2_ [47]. All cultures were further incubated at 37°C for 24 hours. Absorbance readings at 600 nm (Bio-Rad Laboratories, Hercules, CA) were taken at specific intervals to assess growth of cells. When W83 controls reached OD_600nm_ = 0.6–0.8, 1 mL of each sample was removed and 10^−6^ dilutions were made with prewarmed BHI broth. An aliquot (100 *μ*L) of each dilution was spread onto prewarmed and reduced BHI agar plates. Colonies were enumerated after 10 days of anaerobic incubation at 37°C. The colony counts were obtained using imaging processing software (Image J).

### 2.5. Statistical Analysis.

Statistical significance for growth curves at 24 hours was determined at *α* = 0.05 using oneway analysis of variance (ANOVA) with Bonferroni’s posttest. Significant differences in colony growth from the control were determined at *α* = 0.05 using two-tailed nonpaired Student’s *t*-test. All statistical analyses were performed using SPSS Statistics Version 22 (IBM SPSS Statistics, Chicago, IL). A *p* ≤ 0.05 was considered to be significant.

## 3. Results and Discussion

**Figure 1:**
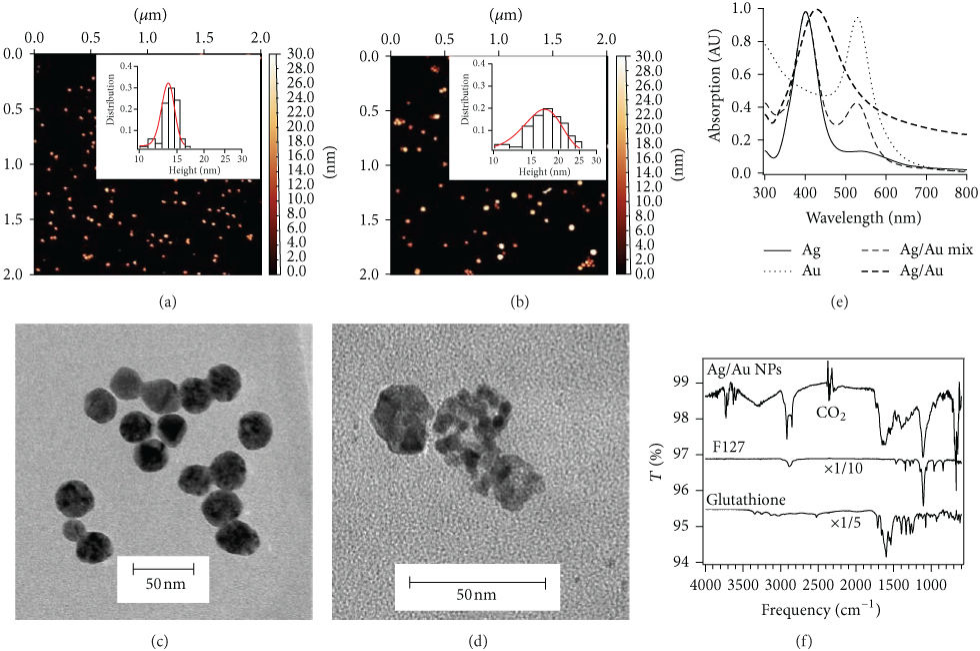
Materials characterization of Ag and Ag/Au nanoparticles. Atomic force microscopy images of (a) Ag (14 ± 2 nm; *N* = 143) and (b) Ag/Au (17 ± 5 nm; *N* = 523) nanoparticles with height distributions. Transmission electron microscopy images of (c) Ag and (d) Ag/Aunanoparticles. The nanoparticles were prepared by adding HAuCl4 (0.2 M, 1 *μ*L) to 1mL of Ag NP seed solution (OD400nm = 12). (e) UV-vis absorption spectra of Ag, Au, a mixture of Ag and Au, and Ag/Au nanoparticles normalized to their peak maxima. (f) Infrared spectra confirming glutathione chemisorption on the surface of Ag/Au NPs. Glutathione (0.1 mM, 10 *μ*L) was added to Ag/Au nanoparticles in 1 mL 5% w/v F127 and left for ≈12 hours. The nanoparticles were washed by centrifugation (10000 ×g; 20 minutes) in water twice before analysis.

Figure 1 shows characterization of Ag/Au NPs synthesized by adding HAuCl_4_ (0.2 M, 1 *μ*L) to 1 mL of Ag NP seed solution (OD_400nm_ ≈ 12). Both synthesized Ag seeds and Ag/Au NPs have a single population with predominantly spherical shapes. Based on the AFM data, the relative heights of the Ag and Ag/Au NPs were determined to be 14 ± 2 and 17 ± 5 nm, respectively. Figures 1(c) and 1(d) are representative TEM images of the Ag seeds and Ag/Au bimetallic NPs, with mean diameters of 22 ± 3 and 16 ± 5 nm, respectively. The darker electron contrast in the Ag/Au NPs indicates heterogeneous electron scattering from the gold and silver. This suggests that the particles have extensive pitting and may have hollow interiors. More importantly, differences in electron contrast in the TEM image indicate that dissolution of Ag(0) and the deposition of Au are not uniform on the NP surface. Galvanic replacement reaction studies indicate that Au deposition preferentially occurs on high-energy {110} and {100} facets [53]. The deposition of Au onto the high-energy facets inhibits Ag oxidation, while the uncoated low energy {111} facets become sites for Ag dissolution in a self-seeding process [54]. This is ultimately responsible for the extensive pitting observed when the bimetallic NPs are prepared. Moreover, the TEM observation that the Ag/Au NPs possess smaller average diameter is indicative of an overall reduction in the number of atoms in the structure of the NPs from alloying at the interfacial regions [55].

Figure 1(e) shows the absorption spectra for Au, Ag, and Ag/Au alloy and a mixture of Ag and Au NPs. The monometallic dispersions of Ag and Au NPs possess localized surface plasmon resonance (LSPR) bands with peak maxima of ≈400 nm and ≈530 nm, respectively. The single LSPR band for the Ag/Au NPs is red shifted (≈428 nm) compared to Ag (≈400 nm) and indicates the formation of alloyed bimetallic NPs [56, 57]. If the NP dispersion had been a mixture of Ag and Au NPs, the LSPR band would be bimodal with peaks corresponding to Ag and Au NPs, respectively. EDS analysis confirmed the incorporation of Au (Au: Ag ratio ≈0.2) (Figure S2). X-ray diffraction characterization showed 30% AgCl contamination on the formed bimetallic NPs when HAuCl_4_ was added to the Ag NPs at 25^∘^C, even after washing by centrifugation [51].

Thermodynamic analysis of bulk silver predicts that Ag(0) is not an equilibrium oxidation state in aqueous environments that are acidic or contain any significant amount of dissolved O_2_ [58]. Under these conditions, Ag^+^ is readily released from bulk silver:

(1) $$4\text{Ag}\left(0\right)+{\text{O}}_{2}\to 2{\text{Ag}}_{2}\text{O}$$

(2) $$2{\text{Ag}}_{2}\text{O}+4{\text{H}}^{+}\to 4\text{A}{\text{g}}^{+}+2{\text{H}}_{2}\text{O}$$

giving an overall stoichiometry of

(3) $$2\text{A}{\text{g}}_{\left(\text{s}\right)}+\frac{1}{2}{\text{O}}_{2}+2{\text{H}}^{+}{}_{\left(\text{aq}\right)}\to 2\text{A}{\text{g}}^{+}{}_{\left(\text{aq}\right)}+{\text{H}}_{2}{\text{O}}_{(\text{l})\cdot }$$

These conclusions also hold true for nanoscale silver [59]. Even though Ag(0) is thermodynamically unstable, the dissolution of Ag(0) to Ag^+^ in Ag colloids is kinetically controlled. Silver ions are also produced during the galvanic replacement of silver by chloroauric acid (HAuCl_4_) to form AgCl during the bimetallic NP synthesis:

(4) $$3\text{A}{\text{g}}_{\left(\text{s}\right)}+\text{Au}{\text{Cl}}_{4}{{}^{-}}_{\left(\text{aq}\right)}\to {\text{Au}}_{\left(\text{s}\right)}+3\text{Ag}{\text{Cl}}_{\left(\text{s}\right)}+{\text{Cl}}^{-}{}_{{\left(\text{aq}\right)}^{\cdot }}$$

There is speciation of the silver ions, with partitioning between aqueous Ag and surface-adsorbed AgCl (AgCl_(s)_ ↔ Ag^+^_(aq)_ +Cl^−^ (aq)), where the silver ions will be sequestered by sulfur (as thiols, e.g., cysteine groups in proteins) and other ligands in biological media [34, 38].

The synthesized Ag/Au NPs were capped with glutathione to increase the colloidal stability of the NPs in saline media (Figure 1(f)). Glutathione was chosen as a capping agent for several reasons. First, glutathione contains thiol groups which have high affinity for noble metal surfaces allowing for the chemisorption of glutathione onto the surface of the Ag/Au NPs [60]. Second, the pH-dependent charged functional groups promote water solubility and interact with biostructures [61, 62]. Lastly, this capping agent can be applied to as-prepared NPs and does not interfere with the antibacterial activity of colloidal Ag NPs [4, 63]. FTIR was used to confirm the chemisorption of glutathione on the surface of the NPs (Figure 1(f)). There is one-to-one correspondence in the fingerprint region below 1700 cm^−1^ between crystalline and glutathione capped Ag/Au NPs with amide I and II bands being observed [64, 65]. Amide I bands between 1600 and 1700 cm^−1^ correspond to the stretching vibrations of the C=O and C-N groups. Amide II bands between 1510 and 1580 cm^−1^ correspond to the in-plane N-H bending, the C-N stretching, and the C-C stretching vibrations. We performed DLS colloidal stability studies with glutathione (0.1 mM) capped and uncapped Ag/Au NPs in 10 mM phosphate saline. The hydrodynamic diameters (~ 60 nm) of glutathione capped Ag/Au NPs were stable for at least 24 hours while the uncapped Ag/Au NPs aggregated after 1 hour (Figure S3). The zeta potentials were ≈−15 meV over the range of F127 capped Ag/Au NP Ag: Au ratios, decreasing to ≈−22 meV when capped with glutathione. The zeta potentials for maltose coated Ag (≈20 meV) and glutathione capped bimetallic NPs are similar indicating that both will be colloidally stable in biological media.

Colloidal stability is also important for antibacterial activity. Bacterial growth over a 24 h period was assessed by measuring the absorbance of the cultures at 600 nm at specific time intervals. Control experiments with 0.1 mM glutathione demonstrate that it does not influence the growth of *P. gingivalis* W83 (Figure S4). Glutathione capped Ag/Au NPs prepared aerobically inhibited the growth of *P. gingivalis* W83 while the uncapped Ag/Au NPs showed no inhibition and precipitated out of solution when exposed to BHI broth overnight (Figure S5). Thus, colloidal stability is crucial for the antibacterial activity of Ag/Au alloy NPs under anaerobic conditions.

**Figure 2:**
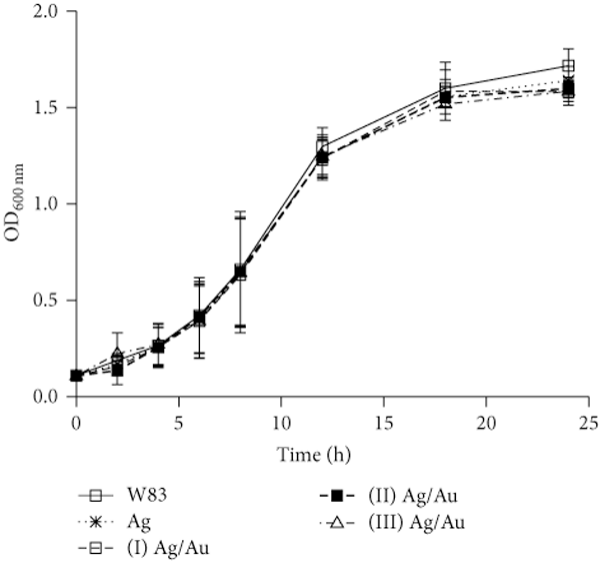
Growth curves of *P. gingivalis* W83 showing the antibacterial activities of anaerobically prepared nanoparticles. Bacterial growth over a 24-hour period was assessed by measuring the absorbance of cultures at 600 nm for specific intervals. *P. gingivalis* W83 was inoculated with anaerobically prepared (≈0.3 nM, 500 *μ*L) glutathione capped Ag and Ag/Au ((I) Au: Ag = 0.10 ± 0.04; (II) Au: Ag = 0.30 ± 0.05; (III) Au: Ag = 2.2 ± 0.1) nanoparticles in 5 mL total volume. Glutathione capping was done using method II. Error bars represent the standard deviation of three experiments.

**Figure 3:**
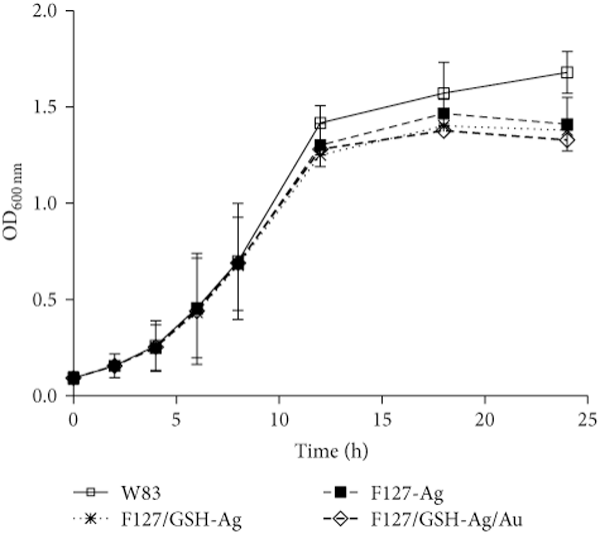
Growth curves of *P. gingivalis* W83 showing the antibacterial activities of aerobically prepared nanoparticles. Bacterial growth over a 24-hour period was assessed by measuring the absorbance of cultures at 600 nm for specific intervals. *P. gingivalis* W83 was inoculated with aerobically prepared Ag nanoparticles coated with F127 (≈0.3 nM, 500 *μ*L) or glutathione capped (≈0.3 nM, 500 *μ*L) and Ag/Au (Au: Ag = 0.30 ± 0.05; ≈0.3 nM, 500 *μ*L) nanoparticles in 5 mL total volume. Glutathione capping was done using method II. Error bars represent the standard deviation of three experiments.

We prepared glutathione capped Ag and Ag/Au (Au: Ag =0.10–2.2) NPs in the anaerobic chamber. *P. gingivalis* W83 was inoculated with these NPs (final concentration ≈ 0.03 nM) for 24 hours. The bacterial growth curves after 24 hours showed no statistical significance between NP treated and untreated bacteria (Figure 2). This result is consistent with previous reports that Ag NP antibacterial activity is from the Ag ions released from partially oxidized silver [38, 66]. Figure 3 shows the growth curves of bacteria incubated with aerobically prepared NPs (final concentration ≈ 0.03 nM). *P. gingivalis* W83 was inoculated with Ag or glutathione capped Ag and Ag/Au (Au: Ag = 0.30 ± 0.05) NPs suspended in 5% aqueous F127. All NPs have similar ~(~25%) inhibition of growth after 24 hours, which is independent of surface coating. This result is consistent with studies showing a weak association of surface capping agents and the rate of Ag^+^ ion release at constant particle core size [10].

Even though Ag NPs show strong antibacterial activity towards aerobes, they exhibit greatly reduced or no antibacterial activity when they are prepared anaerobically. The release of Ag^+^ from the Ag_2_O layer is facilitated by the bacterial proton motive force decreasing the local pH (Equation (3)) [67]. This hypothesis is supported by the data in Figures 2 and 3 demonstrating that partial silver oxidation was necessary for antibacterial activity. This suggests that the oxide layer is necessary for antibacterial activity rather than the surface AgCl, which is present in both aerobically and anaerobically prepared NPs. Thus, the release of Ag^+^ from the Ag_2_O layer may be primarily responsible for the antibacterial activity of Ag NPs [7].

**Figure 4:**
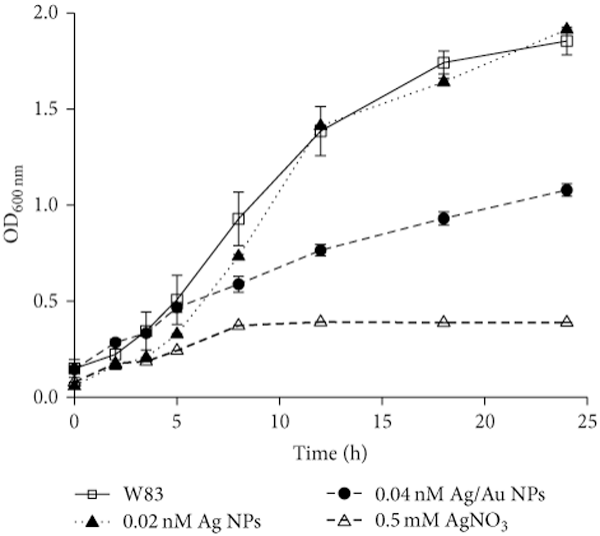
Growth curves of *P. gingivalis* W83 comparing the antibacterial activity of aerobically prepared nanoparticles with 0.5 mM AgNO_3_. Bacterial growth over a 24-hour period was assessed by measuring the absorbance of cultures at 600 nm for specific intervals. *P. gingivalis* W83 (OD_600nm_ ≈ 0.15) was challenged with water (400 *μ*L), maltose coated Ag (≈0.2 nM, 400 *μ*L; OD_λmax_ = 1) nanoparticles, or 0.5 mM AgNO (6.25 mM, 400 *μ*L) in 5 mL total volume. Glutathione capping was done using method I. Error bars represent the standard deviation of three experiments.

Figure 4 shows growth curves at higher concentrations of aerobically prepared glutathione capped Ag/Au NPs (Au: Ag ≈ 0.2; 0.04 nM final concentration). In this case, maltose capped Ag (≈0.02 nM final concentration) and glutathione capped Ag/Au NPs were added from OD_λmax_ = 1 stock solutions. Maltose capped Ag NPs were used to determine if their oxidation is the main determinant in the antibacterial activity of Ag/Au NPs. The growth curve of *P. gingivalis* W83 is shown for comparison. Aerobically prepared Ag/Au NPs as well as 0.5 mM AgNO_3_ exhibited antibacterial activity against *P. gingivalis* W83 at 12 (*p* < 0.01) and 24 (*p* < 0.01) hours. Below 0.1 mM AgNO_3_, the bacteria recover from the inhibitory effects of Ag^+^ affer 24 hours (Figure S6). Indeed, a closer examination of Figure 4 reveals that maltose capped Ag NPs prepared aerobically have an inhibitory effect on bacterial growth during the early exponential growth phase (4–12 hours).

The sensitivity of *P. gingivalis* W83 was also assessed using colony count assays when cultures were at OD ≈ 0.5 (after six hours of treatment; ≈8 × 10^8^ CFU/mL W83 control) (Table 1). The glutathione capped OD_λmax_ = 1 (≈0.04 nM) and OD_λmax_ = 4 (≈0.14 nM) Ag/Au NPs (Au: Ag ≈ 0.2) decreased *P. gingivalis* W83 survival by approximately log (0.4) 10 CFU/mL (≈3-fold decrease) and log_10_ (1.3) CFU/mL (≈20-fold decrease), respectively (*p* < 0.01). Collectively, these data indicate that the Ag/Au NPs (>0.04 nM) have comparable antibacterial activities to 0.5 mM AgNO_3_. The preparation process for Ag/Au NPs involves the Ag NPs being centrifuged on the bench to remove the excess reagents and suspended in 5% w/v F127 aqueous solution prior to adding Au(III). During this process to synthesize Ag/Au NPs, further oxidation of the silver occurs and this enhances its antibacterial activity.

**Figure 5:**
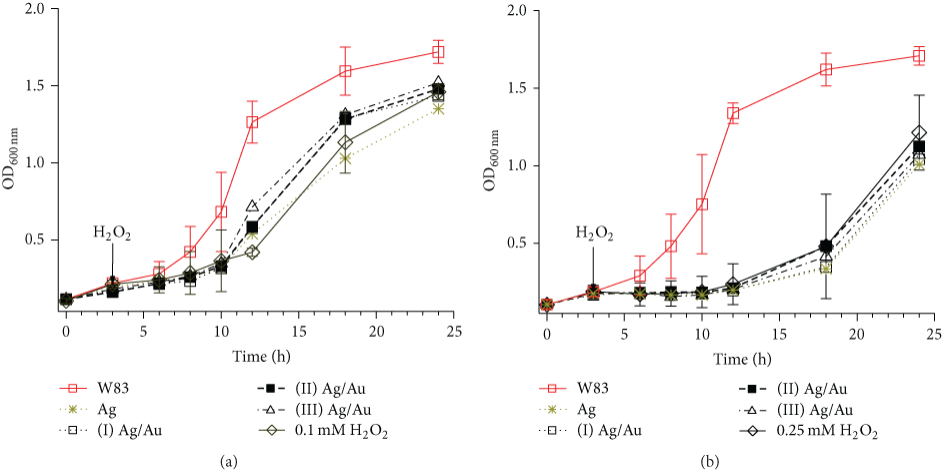
Growth curves of *P. gingivalis* W83 stressed with (a) 0.1 or (b) 0.25 mM H_2_O_2_ in the presence of anaerobically prepared Ag and Ag/Au nanoparticles. Bacterial growth over a 24-hour period was assessed by measuring the absorbance of cultures at 600 nm for specific intervals. Bacteria were inoculated with glutathione (≈0.3 nM, 500 *μ*L) capped Ag and Ag/Au ((I) Au: Ag = 0.10±0.04; (II) Au: Ag = 0.30±0.05; (III) Au: Ag = 2.2±0.1) nanoparticles in 5 mL total volume. Glutathione capping was done by method II. Hydrogen peroxide was added when OD_600nm_ of W83 control was ≈0.2. Error bars represent the standard deviation of three experiments.

**Figure 6:**
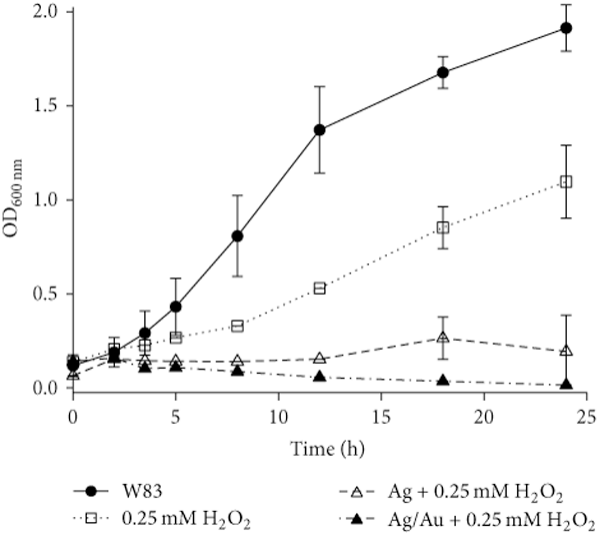
Growth curves of *P. gingivalis* W83 stressed with 0.25 mM H_2_O_2_ in the presence of aerobically prepared Ag and Ag/Au nanoparticles. Bacterial growth over a 24-hour period was assessed by measuring the absorbance of cultures at 600 nm for specific intervals. Actively growing *P. gingivalis* W83 (OD_600_ ≈ 0.2) was challenged with maltose coated Ag (≈0.2 nM, 400 *μ*L; OD_λmax_ = 1) or glutathione capped Ag/Au (Au: Ag ≈ 0.2; ≈0.4 nM, 400 *μ*L; OD_λmax_ = 1) nanoparticles in 5 mL total volume. Glutathione capping was done by method II. Hydrogen peroxide was added when OD_600nm_ of W83 control was ≈0.2. Error bars represent the standard deviation of three experiments.

Because it is well established that periodontal disease is associated with inflammation [21, 22], we assessed the influence of hydrogen peroxide on the antibacterial activities of the NPs (Figures 5 and 6). We grew *P. gingivalis* W83 culture overnight. Prewarmed BHI broth was inoculated using the overnight culture to an OD ≈ 0.1. Bacteria were grown to OD ≈ 2 and then split into equal aliquots and inoculated with anaerobically prepared NPs and stressed with subinhibitory(0.1 mM) or inhibitory (0.25 mM) concentrations of H_2_O_2_ [47]. All cultures were further incubated at 37°C for 12 or 24 h (Figure 5). We observed that the addition of anaerobically prepared NPs to *P. gingivalis* W83 exposed to H_2_O_2_ did not further inhibit bacterial growth. In contrast, aerobically prepared Ag and Ag/Au NPs in combination with 0.25 mM of H_2_O_2_ completely inhibited bacterial growth (Figure 6).

To further assess the influence of H_2_O_2_ concentration (0.01–0.25 mM) on aerobically prepared NP antibacterial activity, we compared bacterial growth at 12 and 24 hours (Figure S7). Overnight cultures of *P. gingivalis* W83 were incubated anaerobically at 37°C until OD_600nm_ ≈ 0.1 and inoculated with treatments of water, maltose capped Ag (≈0.02 nM), or glutathione capped Ag/Au (Au: Ag ≈ 0.2) nanoparticles. When OD_600nm_ of the *P. gingivalis* W83 control was ≈ 0.2, H_2_O_2_ was added. Only at 0.25 mM H_2_O_2_ were there synergistic growth inhibition effects on *P. gingivalis* W83 with AgNO_3_, Ag, and Ag/Au NPs (Figures S7A, S7C). To correlate the effect of H_2_O_2_ and NPs, the absorbance measurements were normalized against the corresponding values in the absence of H_2_O_2_ after 12 and 24 hours (Figures S7B, S7D). We note that AgNO_3_ has the best antibacterial activity after 24 hours. A likely explanation is that the molar excess of H_2_O_2_ oxidizes the *in situ* generated Ag NPs, made in the reducing environment of the growth media, back to Ag^+^.

**Figure 7:**
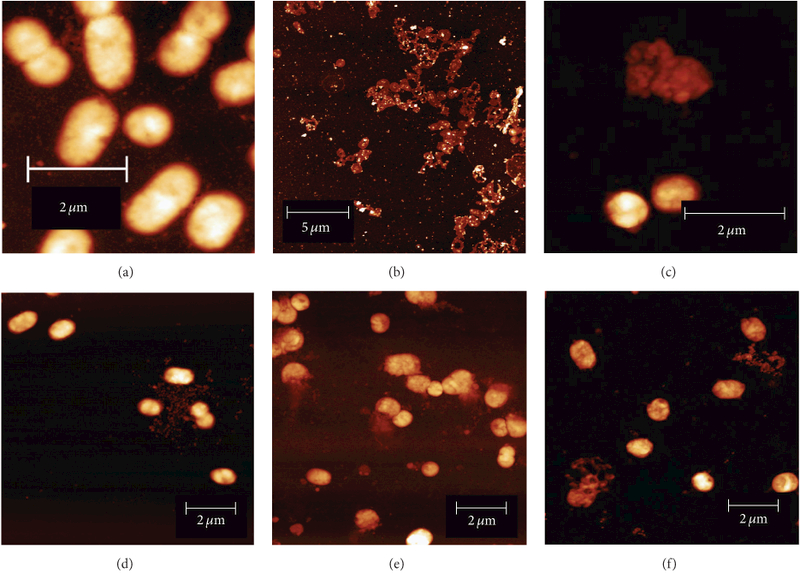
Representative atomic force microscopy height images of *P. gingivalis* W83 exposed to aerobically prepared Ag/Au nanoparticles. (a) Cells dividing in the exponential growth phase with undamaged membranes and typical dimensions (~1 × 1 *μ*m^2^). (b) *P. gingivalis* W83 exposed to 0.5 mM AgNO for ≈33 hours (corresponding to OD ≈ 0.5 in W83 control growth phase) revealed significant bacterial lysis and membrane disruption. (c) *P. gingivalis* W83 exposed to 0.25 mM H_2_O_2_ showing bacterial lysis. (d) *P. gingivalis* W83 exposed to glutathione capped Ag/Au nanoparticles for ≈10 min is similar to untreated bacteria and displays no signs of structural damage. (e) When *P. gingivalis* W83 is exposed to glutathione capped Ag/Au nanoparticles for ≈5 hours the majority of the bacterial membranes are completely disrupted with intracellular material leaking out. (f) *P. gingivalis* W83 exposed to glutathione capped Ag/Au nanoparticles in the presence of 0.25 mM H_2_O_2_

As Ag NPs are capable of disrupting microbial cell walls and bacterial membranes [68, 69], we used AFM to investigate the action of aerobically prepared glutathione capped Ag/Au NPs on the surface structure of *P. gingivalis* W83. The AFM images of untreated *P. gingivalis* W83 reveal intact cells with undamaged membranes and typical dimensions of ~1 μm (Figure 7(a)). In contrast, AFM images of *P. gingivalis* W83 exposed to 0.5 mM AgNO_3_ for ≈3 hours resulted in cell lysis and death (Figure 7(b)). The bright contrast in the image is assigned to Ag NPs formed from the reduction of Ag^+^ ions. Broth containing *P. gingivalis* W83 will be strongly reducing, containing volatile sulfur compounds including hydrogen sulfide, methyl mercaptan (methanethiol), and dimethyl sulfide, where methyl mercaptan is believed to play a role in the pathogenicity of *P. gingivalis* W83 [70]. Thus, it is likely that Ag^+^ ions are reduced by thiols such as methyl mercaptan:

(5) $$2{\text{Ag}}^{+}{}_{\left(\text{aq}\right)}+2{\text{CH}}_{3}{\text{SH}}_{\left(\text{aq}\right)}\to {\text{CH}}_{3}{\text{SSCH}}_{3(\text{aq})}+2{\text{H}}^{+}{}_{\left(\text{aq}\right)}+2{\text{Ag}}_{(\text{s}),}$$

which have lower standard electrode potentials than silver [71]. Hydrogen peroxide, like silver nitrate, will also cause significant membrane damage (Figure 7(c)). AFM images of *P. gingivalis* W83 exposed to glutathione capped Ag/Au NPs for ≈10 min are similar to untreated bacteria and display no signs of structural damage (Figure 7(d)). When *P. gingivalis* W83 was exposed to glutathione capped Ag/Au NPs for ≈5 hours, AFM images show that a majority of the bacterial membranes are completely disrupted and that intracellular material is leaking out (Figure 7(e)). This indicates that Ag/Au NP inhibition acts in a time-dependent manner. Complete membrane disruption is achieved in the presence of both 0.25 mM hydrogen peroxide and Ag/Au NPs (Figure 7(f)).

## 4. Conclusions

We describe a facile, economic, and environmentally benign method for the synthesis of glutathione capped Ag/Au bimetallic NPs. The synthesized NPs of Au: Ag ratio ≈ 0.2 capped with glutathione were colloidally stable in saline solution for at least 24 hours. The aerobically prepared glutathione capped Ag and Ag/Au (Au: Ag ratio ≈ 0.2) NPs have similar antibacterial activities against the anaerobic oral pathogen *P. gingivalis*. Moreover, the antibacterial activity of aerobically prepared NPs is enhanced in the presence of hydrogen peroxide. These data support the idea that partially oxidized silver with subsequent Ag^+^ ion release from the Ag_2_O overlayer is necessary for antibacterial activity [10, 37,38]. There are compelling reasons for using Ag/Au bimetallic NPs. First, as a reservoir for Ag^+^, the Ag/Au NPs stored in aqueous F127 copolymer solutions are more amenable for long-term storage. Second, Ag/Au NPs have the potential of providing a larger antimicrobial therapeutic window whilst minimizing acute toxicity to host eukaryotic cells. As a future direction, *in vitro* and *in vivo* cytotoxicity studies are needed to quantify the efficiency and toxicity of these biocompatible Ag/Au NPs.

## Supplementary Material

Supplement

## Figures and Tables

**Table 1:** Antibacterial activity on *P. gingivalis* W83 determined by colony forming units.

	log_10_ (CFU/mL)	^a^Δlog_10_ (CFU/mL)
W83	9.4	0
Maltose -Ag NPs	9.5	+0.1
GSH-Ag/Au NPs (0.04 nM)	9.0	−0.4
GSH-Ag/Au NPs (0.14 nM)	8.1	−1.3
0.25 mM H_2_O_2_	7.9	−1.5
0.25 mM H_2_O_2_ + Ag/Au (0.04 nM)	7.8	−1.6
BHI	0.0	0

a Negative sign: log CFU/mL decrease.
