# Supplementary material for: Antibacterial Activity of Partially Oxidized Ag/Au Nanoparticles against the Oral Pathogen Porphyromonas gingivalis W83
Source: J Nanomater. Author manuscript; Available in PMC 2018 Sep 20. (PMC6146971; doi:10.1155/2016/9605906)
Supplement: Supplement [file NIHMS942705-supplement-Supplement.pdf]

Supplementary Information

Antibacterial Activity of Partially Oxidized Ag/Au Nanoparticles against the Oral Pathogen *Porphyromonas gingivalis* W83

Megan S. Holden[1], Jason Black[2], Ainsely Lewis[2], Marie-Claire Boutrin[3], Elvin Walemba[4], Theodore S. Sabir[5], Danilo S. Boskovic[1,4], A. Wilson Aruni[3], Hansel M. Fletcher[3], and Christopher C. Perry[1*]

[1]Loma Linda University School of Medicine, Division of Biochemistry, Loma Linda, California 92350

[2]Northern Caribbean University, Manchester, Jamaica

[3]Loma Linda University School of Medicine, Division of Microbiology and Molecular Genetics, Loma Linda, California 92350

[4]Loma Linda University School of Medicine, Department of Earth and Biological Sciences, Loma Linda, California 92350

[5]Faulkner University, College of Arts and Sciences, Montgomery, Alabama, 36109

Running title: Antibacterial activity of partially oxidized Ag/Au nanoparticles

A. Material Characterization

Instrumentation. UV-Vis spectra of nanomaterials were recorded using a Varian Cary 300 spectrophotometer equipped with a temperature controller. All UV-Vis measurements were made using a quartz cell with a 1 cm path length at 25 ^{o}C.

A Thermo NNS Energy-dispersive X-ray (EDX) analyzer attached to a Vega LSH scanning electron microscope was used to determine the composition of the Ag/Au bimetallic nanoparticles (NPs). EDX measurements were performed on at least five different regions on two different samples.

Atomic force microscopy (AFM) images were generated with a multimode 8 scanning probe microscope (Bruker, Santa Barbra CA) in the peak force tapping (k = 0.4 Nm^{-1}, f = 70 kHz) mode as previously described [1, 2]. Briefly, NPs were prepared for AFM measurements by removing excess reagents (samples were centrifuged at 10,000 × *g* for 15 min and suspended in water twice) followed by dropping 50 µL of solution onto a freshly prepared parafilm. A silanized (0.1% w/v) 3-Aminopropyl-triethoxysilane (APTES) 18 mm mica disk (Ted Pella, Redding CA) was then applied onto the sample facedown to obtain spreading for at least 10 minutes. The mica disk was then rinsed with ethanol and water prior to imaging. To prepare *P. gingivalis* W83 for imaging, the bacteria were grown to early exponential phase before being exposed to glutathione capped Ag/Au NPs (OD = 1) for 10 min or 5 hours. The bacteria (1 mL) were then washed by centrifugation at 2,500 × *g* for 5 min and suspended in 10 mM phosphate-buffered saline (1 mL final volume). The resuspended bacteria (100 µL) were further diluted to 1 mL in 10 mM phosphate-buffered saline. The resulting bacterial suspension (40 µL) was dried onto a silanated mica disk under nitrogen. Samples were rinsed well with water and allowed to dry prior to same day imaging. All microscopy (TEM and AFM) measurements were processed using the Gwyddion analysis tool (downloadable from http://gwyddion.net).

Digital transmission electron microscopy was carried out on a Philips Tecnai 12 instrument operating at 80 kV fitted with a Gatan camera. Samples were prepared for electron microscopy measurements by removing excess reagents (samples were centrifuged at 10,000 × *g* for 15 min and resuspended in water twice) followed by dropping 5 to 10 µL of solution onto a silanated 200 mesh carbon-coated Cu grid (Ted Pella, Redding CA). Samples were allowed to air dry.

Fourier transform infrared spectroscopy (FTIR) was carried out using a Jasco FTIR 4100 with a deuterated triglyceride sulfate detector. In order to obtain the FTIR spectra a drop of the nanoparticle solution was placed on the surface of an attenuated total reflectance germanium crystal and dried under nitrogen. Spectra were obtained at 4 cm^{-1} resolution and 64 scans were obtained for statistical averaging.

Dynamic light scattering (DLS) was used to evaluate the colloidal stability of glutathione capped and uncapped Ag/Au alloy NPs. A Nicomp™ 380 XLS Zeta Potential/Particle Sizer (PSS Nicomp, USA) equipped with a He-Ne laser wavelength 638 nm and a power output of 60 mW was used for all DLS experiments. Briefly, 240 µL of glutathione

capped or uncapped Ag/Au NPs (OD = 1) were suspended to a final volume of 3 mL in 10mM phosphate buffered saline (pH 7.5). Volume weighted DLS was used to evaluate nanoparticle aggregation immediately after dilution as well as 1, 2, 4, 6, 8, and 24 hours after dilution. All data were collected at 25 °C and at a scattering angle of 168.6° with a square acrylic cuvette (3 mL volume). Refractive index of water and viscosity were assumed to be 1.33 and 8.9×10^{-4} Ns m^{-2}, respectively.

References

1. T. S. Sabir, D. Yan, J. R. Milligan, A. W. Aruni, K. E. Nick, R. H. Ramon, J. A. Hughes, Q. Chen, R. S. Kurti and C. C. Perry, "Kinetics of Gold Nanoparticle Formation Facilitated by Triblock Copolymers," *The Journal of Physical Chemistry C*, vol. 116, no. 7, pp. 4431-4441, 2012.
2. T. S. Sabir, L. K. Rowland, J. R. Milligan, D. Yan, A. W. Aruni, Q. Chen, D. S. Boskovic, R. S. Kurti and C. C. Perry, "Mechanistic Investigation of Seeded Growth in Triblock Copolymer Stabilized Gold Nanoparticles," *Langmuir*, vol. 29, no. 12, pp. 3903-3911, 2013.

B. Supplemental Figures

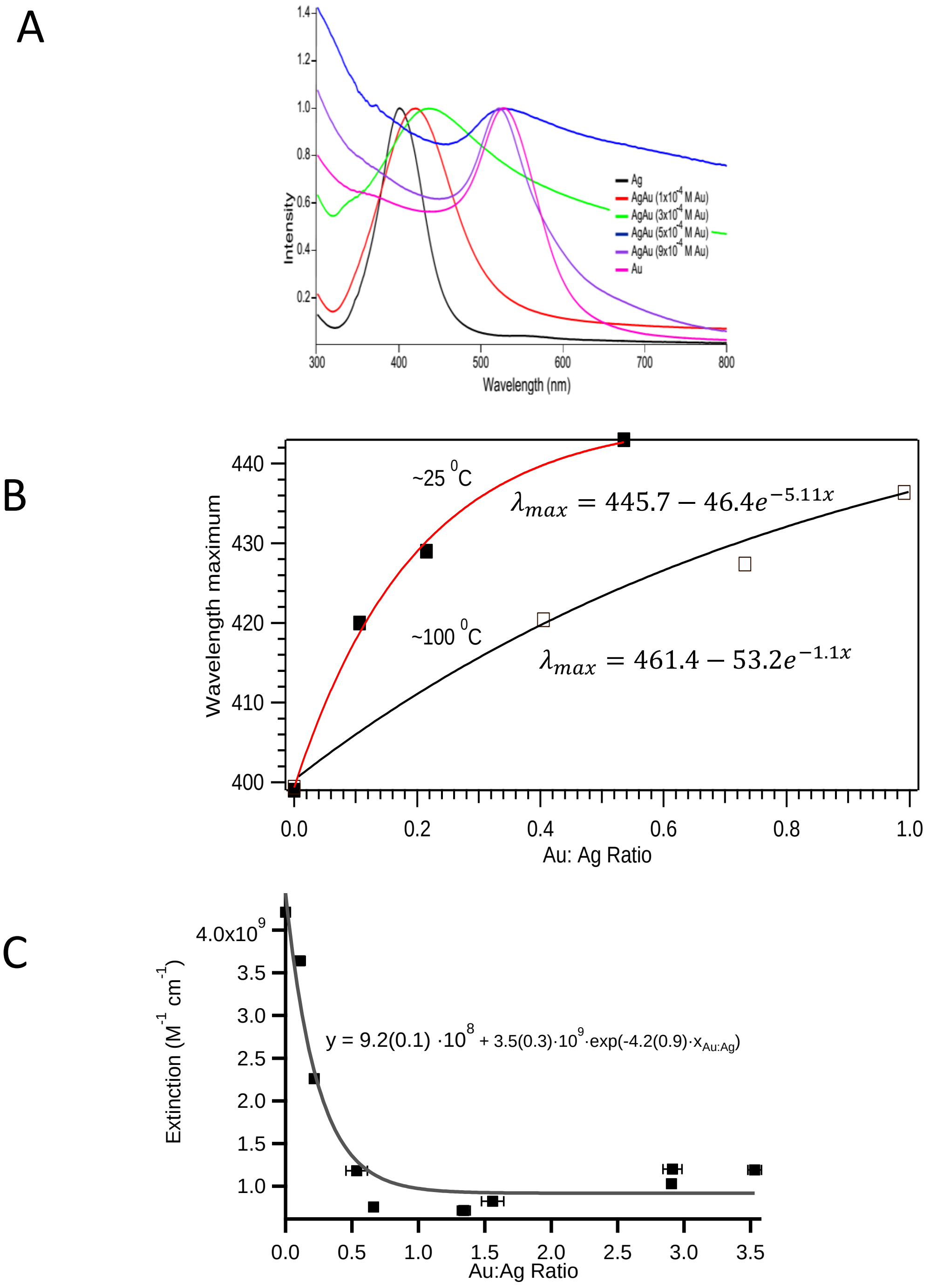

Figure S1. Spectroscopic determination of Au:Ag ratio and nanoparticle concentration. (A) UV-vis spectra of Ag/Au nanoparticles normalized to the peak maxima. (B) A typical plot of wavelength maxima against Au:Ag ratio for bimetallic nanoparticles synthesized at 25 and 100 °C. (C) Plot of extinction coefficient for ~ 20 nm NPs against Au:Ag ratio.

A

Element	Weight %	Atomic %
Ag-L	78.87	87.20
Au-L	21.13	12.80
Totals	100.00	100.00

B

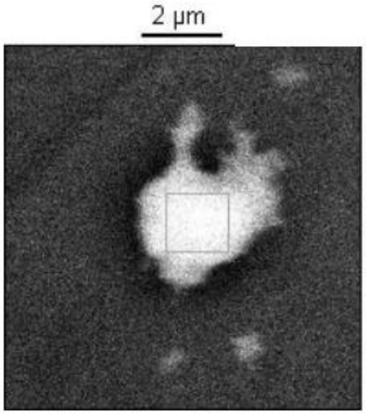

C

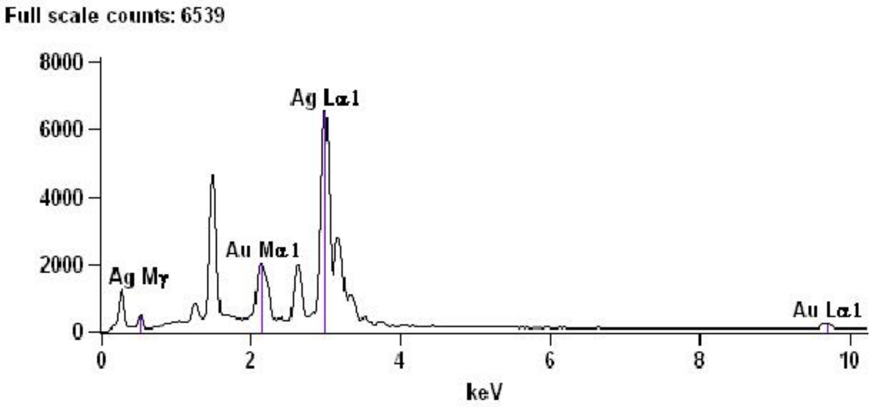

Figure S2. Typical EDX analysis data set for Ag/Au NPs. Data are (A) Elemental composition table (calculated exclusively by fitting silver and gold contributions), (B) SEM image of the analyzed zone, and (C) EDX spectrum for Ag/Au NPs.

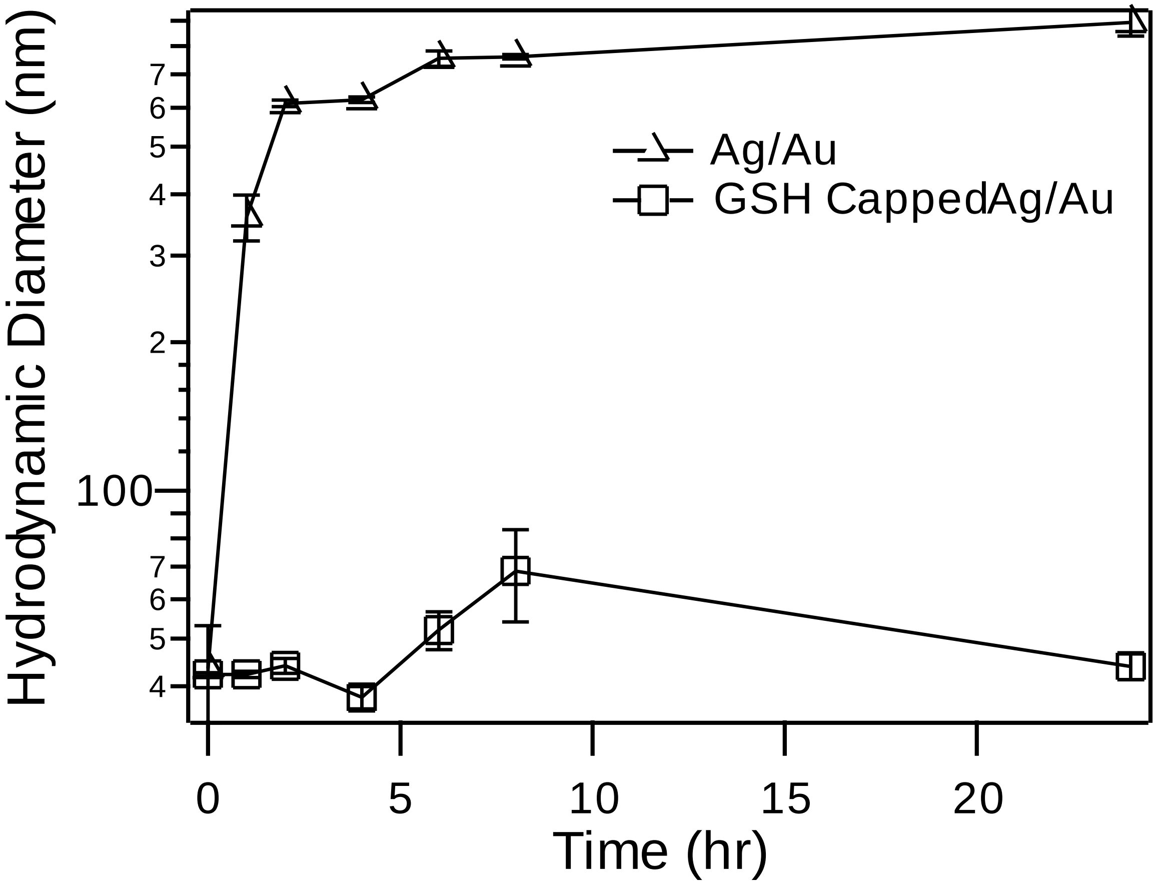

Figure S3. Capping and colloidal stability of Ag/Au with glutathione. DLS data depicting the aggregation of glutathione capped and uncapped Ag/Au NPs in 10 mM phosphate buffer saline solution (138 mM NaCl, 3 mM KCl) against time.

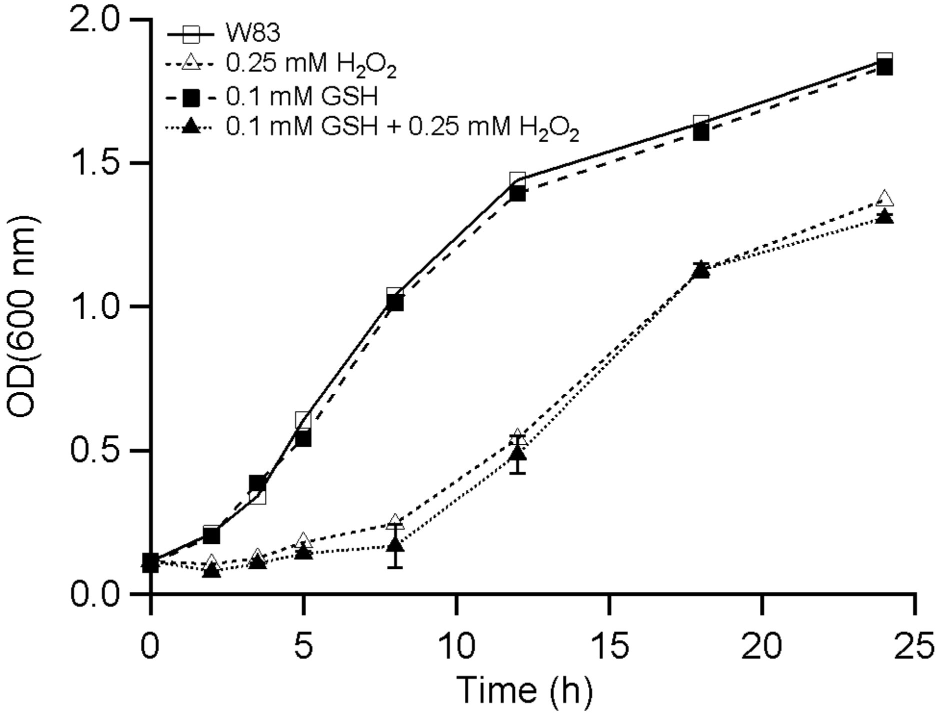

Figure S4. Growth curves of *P. gingivalis* W83 showing the effect of 0.1 mM glutathione in the absence or presence of 0.25 mM H_2O_2. The total solution volume is 5 mL. Bacterial growth over a 24 hour period was assessed by measuring the absorbance of the cultures at 600 nm at specific time intervals. Error bars represent the standard deviation of three experiments.

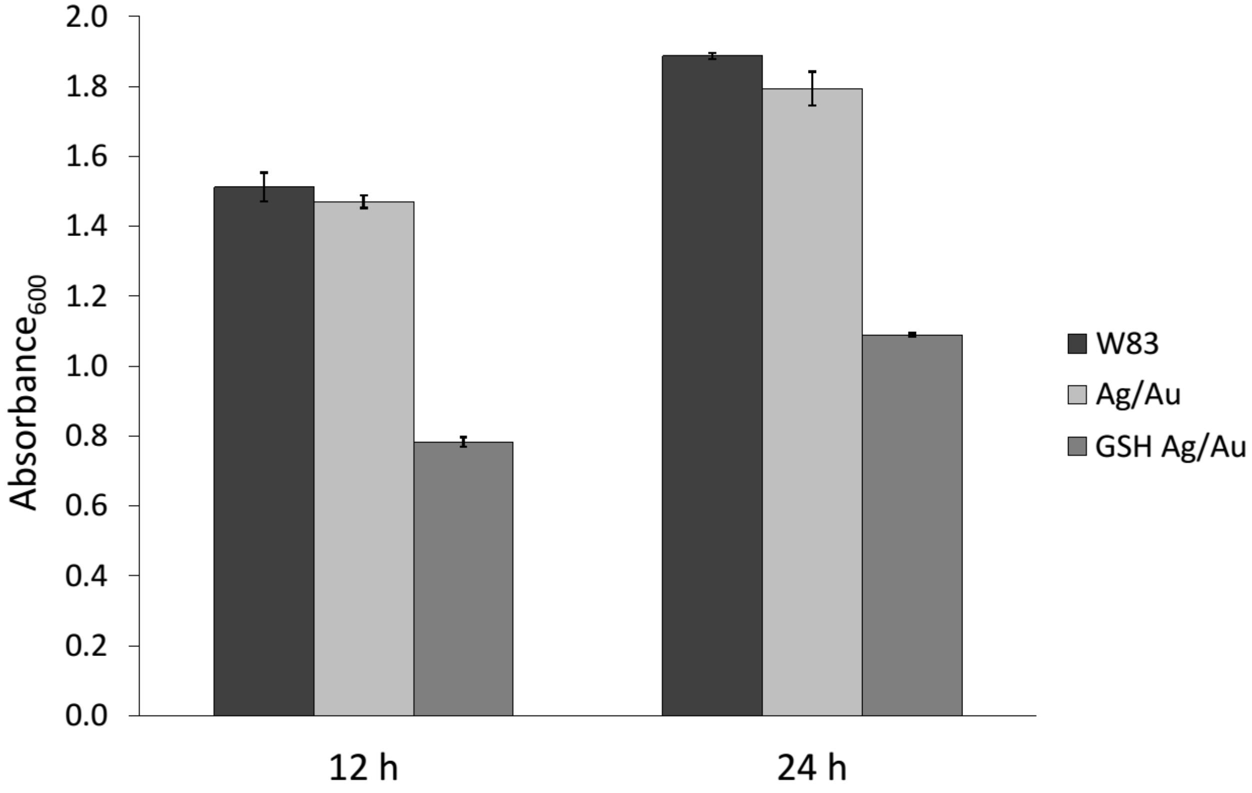

Figure S5. Effect of glutathione capped and uncapped Ag/Au nanoparticles on *P. gingivalis* W83 growth at 12 and 24 hours. *P. gingivalis* W83 was exposed to water (control), uncapped Ag/Au NPs, or glutathione capped Ag/Au NPs. The absorbance at 600 nm was measured at for 12 and 24 h after incubation at 37 °C under anaerobic conditions to assess bacterial growth. Glutathione capping was done using method I. Error bars are standard deviation.

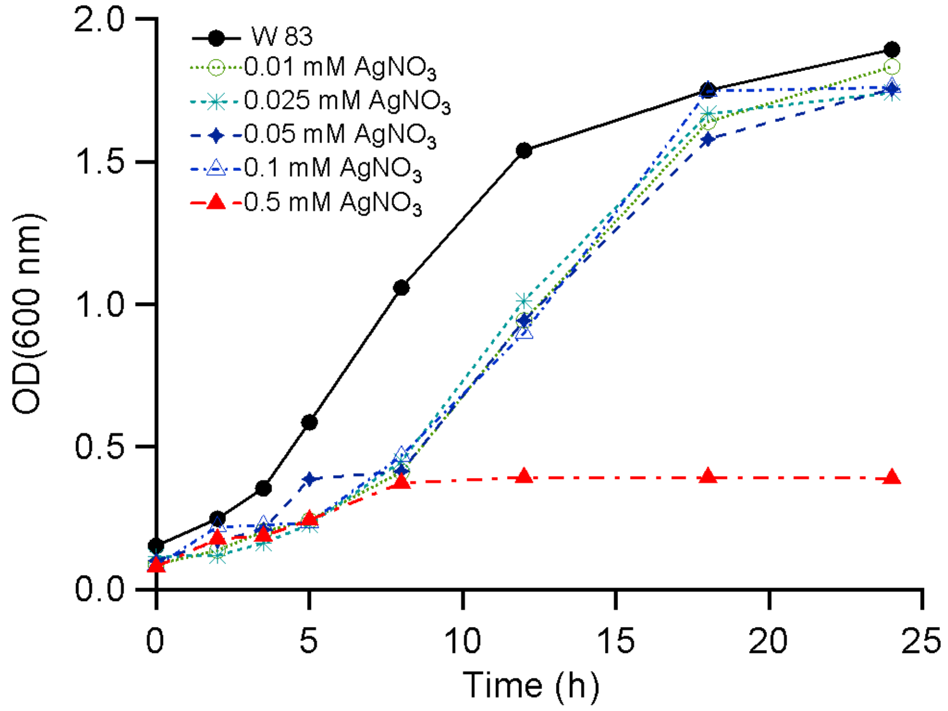

Figure S6. Representative growth curves of *P. gingivalis* W83 following exposure to $AgNO_3$. In each case, 400 mL of $AgNO_3$ was used to give final concentrations in 5 mL total volume: 0.01 mM (0.125, mM stock) 0.025 mM (0.3125 mM stock), 0.05 mM (0.625 mM stock), and 0.1 mM (1.25 mM stock) and 0.5 mM (6.25 mM stock).

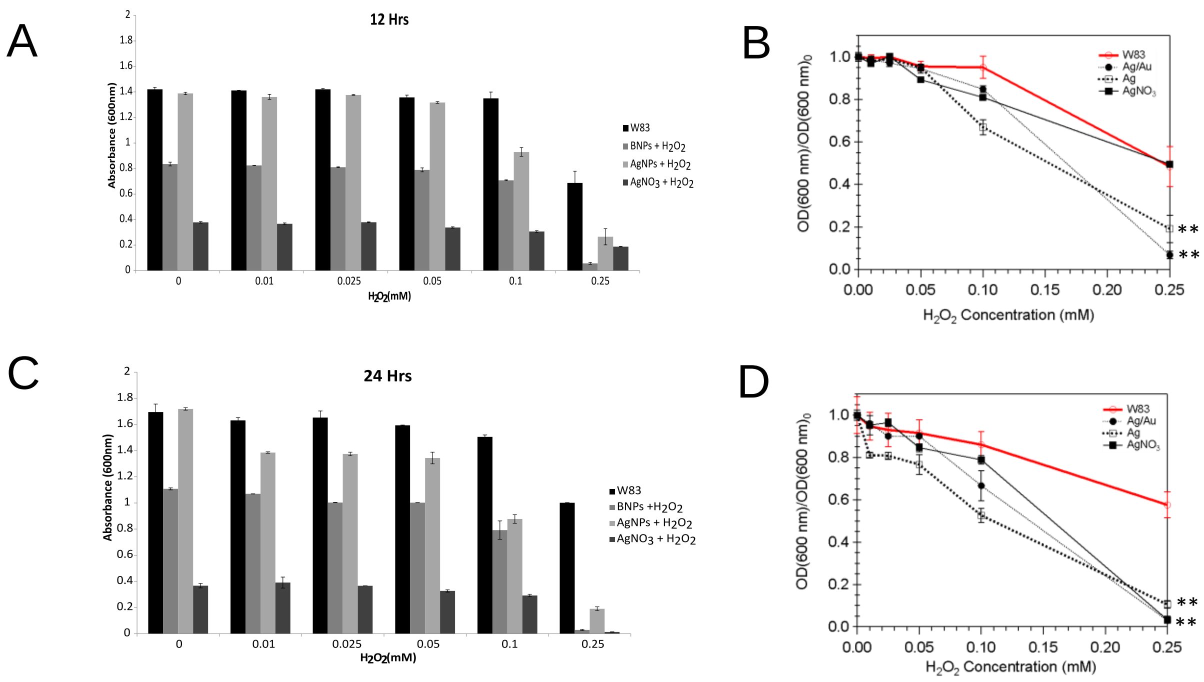

Figure 7. Effect of H_2O_2 in combination with nanoparticless prepared aerobically at 12 and 24 hours. Aliquots of overnight *P. gingivalis* W83 cultures (100 μL) were used to inoculate 4.6 mL aliquots of BHI, incubated anaerobically at 37 °C until $OD_{600\ nm} \approx 0.1$, and inoculated with 400 μL of water, maltose capped Ag (≈ 0.2 nM, 400 μL; $OD_{\lambda max} = 1$), or glutathione capped Ag/Au (Au:Ag ≈ 0.2;≈ 0.4 nM, 400 μL; $OD_{\lambda max} = 1$) nanoparticles. Glutathione capping was done using method I. When the $OD_{600\ nm}$ of the W83 control was ≈ 0.2, H_2O_2 was added. (A) Bar plots of the absorbances after 12 hours incubation of *P. gingivalis* W83 with Ag, Ag/Au nanoparticles and $AgNO_3$ against various concentrations of H_2O_2. (B) Absorbances normalized against the corresponding values in the absence of H_2O_2 after 12 hour incubation of *P. gingivalis* W83 treated with Ag, Ag/Au, or $AgNO_3$. (C) Bar plots of the absorbances after 24 hours incubation of *P. gingivalis* W83 with Ag, Ag/Au nanoparticles and $AgNO_3$ against the concentrations of H_2O_2. (D) Absorbances normalized against the corresponding values in the absence of H_2O_2after 24 hour incubation of *P. gingivalis* W83 treated with Ag, Ag/Au, or $AgNO_3$. Error bars represent standard deviation, $^{**}P<0.01$ one-way ANOVA- Bonferroni analysis. All absorbances were measured at 600 nm.
